# Endothelial to mesenchymal transition (EndMT): an active process in Chronic Obstructive Pulmonary Disease (COPD)?

**DOI:** 10.1186/s12931-016-0337-4

**Published:** 2016-02-22

**Authors:** Sukhwinder Singh Sohal

**Affiliations:** School of Health Sciences, University of Tasmania, Locked Bag – 1322, Newnham Drive, Launceston, TAS 7248 Australia; Breathe Well Centre of Research Excellence for Chronic Respiratory Disease and Lung Ageing, School of Medicine, University of Tasmania, Hobart, 7000 Australia

**Keywords:** Epithelial mesenchymal transition (EMT), Small airways, Large airways, COPD, Lung cancer, Endothelial to mesenchymal transition (EndMT), Airway remodelling

## Abstract

Small airway fibrosis is the main contributor to physiological airway dysfunction in COPD. One potential mechanism contributing to small airway fibrosis is epithelial mesenchymal transition (EMT). When associated with angiogenesis (so called EMT-Type-3) it may well also be the link with the development of airway epithelial cancer, which is closely associated with COPD and predominantly in large airways. In a recent study published in *Respiratory Research*, Reimann and colleagues, showed increased expression of S100A4 in vasculature of human COPD and murine lungs. It is quite possible that the process of endothelial to mesenchymal transition (EndMT) is active in COPD lungs which we wish to comment on.

## Background

**Fig. 1:**
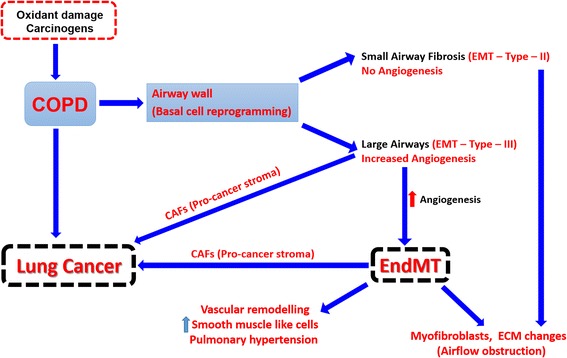
Potential contribution of epithelial mesenchymal transition (EMT) and endothelial mesenchymal transition (EndMT) to pathogenies of COPD and its linkage to lung cancer through formation of pro-cancer stroma

COPD is mainly caused by smoking, at least in the western countries and presents with shortness of breath that is progressively irreversible and associated with an abnormal inflammatory response of the airways in response to noxious particles and gases [1]. It is a worldwide health problem and the fourth most common cause of chronic disability and mortality, even in developed countries. Unfortunately, the research effort directed into this has been disproportionately weak compared to its clinical and scientific importance, and indeed COPD itself is the least researched of all common chronic conditions compared to its social importance. The term “chronic obstructive pulmonary disease” (COPD) now widely used, was first introduced into the literature in 1964 [2]. Later on in the 1970s and 1980s, sub-phenotypes such as emphysema, chronic bronchitis, chronic obstructive bronchitis and chronic bronchitis with emphysema were used [3]. It is a complex disease, and can have both airway and lung parenchymal components involved. Pathologically, it involves structural changes in lung parenchyma, airways, vessels [4]. Remodelling in COPD, may occur in response to smoking-induced damage to the lungs, but the details of structural changes and underlying mechanism are poorly described or understood [4]. One potential mechanism contributing to small airway fibrosis/obliteration and epithelial cancers in COPD is epithelial mesenchymal transition (EMT) [5–9]. Vascular remodelling has also been widely reported in COPD both in mild to severe disease but the mechanisms behind, again are poorly understood [10–13]. Recent study by Reimann and colleagues published in *Respiratory Research*, highlighted vascular remodelling in COPD with increase in S100A4 expression (or FSP-1, fibroblast specific protein) in vasculature of human COPD and murine lungs [14]. Authors discussed the importance of vascular remodelling in pathophysiology of COPD, however there is no clear information on how S100A4 might be contributing to the vascular remodelling in COPD. It is quite possible that the process of endothelial to mesenchymal transition (EndMT) is active in COPD lungs (Fig. 1).

## Main text

The classically described process of EMT involves phenotypic change and migration of epithelial cells into the sub-epithelial mesenchyme in the lamina propria (LP) to function as extracellular-matrix producing fibroblasts/myofibroblasts [15–19]. EMT is a vital process during embryogenesis (EMT-Type-I), but can also be induced as a result of persistent damage and tissue inflammation [20–22]. There are then two subsequent outcome possibilities with active EMT: severe and even complete organ fibrosis (EMT-Type-II), development of a pre-malignant stroma when associated with angiogenesis (EMT-Type-III) [10, 13, 15–22].

We recently published that EMT is an active process in both small and large airways of COPD patients [5, 8, 9]. Furthermore, the reticular basement membrane (Rbm) in large airways is hyper-vascular [10] i.e., give the appearance of active EMT-Type-III, and of course it is the large airways in COPD, where cancer formation is common (Fig. 1), especially squamous cell carcinoma [15, 21, 23]. In small airways no hyper-vascularity of the Rbm was observed [5], indicating that EMT-Type-II is active and contributing to small airway fibrosis and obliteration at this site, [5, 16–19]. Recently in a randomized controlled trial, we also reported that inhaled corticosteroid fluticasone propionate given over six months suppressed EMT-related changes in large airways of COPD patients [24]. This was the first study reporting anti-EMT effects of inhaled corticosteroids in COPD.

Similar to the process of EMT is endothelial to mesenchymal transition (EndMT), in which endothelial cells lose their adhesion properties and apical-basal polarity to form highly invasive, migratory, spindle-shaped, elongated mesenchymal cells (fibroblasts/myofibroblasts) and contribute to different pathological processes in the organism in a number of ways [25]. EndMT is a critical process during embryogenesis, especially in embryonic cardiac development [26]. However, in response to persistent damage and inflammation, EndMT can lead to complete organ fibrosis [27] and cancer as well [25, 27–30]. In malignancy, it is suggested that myofibroblasts or cancer associated fibroblasts (CAFs) produced during EndMT can facilitate tumour growth and cancer progression, which fits with the underlying pathology as the tumour tissue is heavily associated with increased angiogenesis so it is quite possible that vascular endothelial cells are contributing to the pool of CAFs (Fig. 1) by transforming into a mesenchymal cell type [28, 30, 31]. EndMT can also initiate the formation of pro-cancer stroma quite similar to EMT-Type-III, so it has the potential for both, to initiate cancer but at the same time helps the tumour to thrive [30].

Vascular remodelling has also been reported in COPD, main structural changes involve intimal and medial thickening, leading to reduction of lumen diameter and muscularization of arterioles [32]. The other changes involve hypo-vascular lamina propria and hyper-vascular Rbm in large airways of smokers and COPD [11, 13, 33–35]. Both loss of vessels and vascular remodelling give rise to pulmonary hypertension in COPD [32, 36]. Interestingly, these vascular remodelling changes are also observed in early COPD and in normal lung function current smokers [11, 13, 14, 32–34, 37]. Reimann and colleagues not only observed the expression of S100A4 in occulated arteries but also in very small vessels with a diameter of less than 50 μm [14]. Abnormal deposition of pulmonary smooth like cells has been considered as the key pathological feature of arterial remodelling [38]. These cells lead to increased production of extracellular matrix proteins, with deposition of collagen and elastin proteins contributing to narrowing of arterial lumen hence pulmonary hypertension. But the origin of these smooth muscle like cells and the underlying mechanisms involved in vascular remodelling are poorly understood [38].

This recent study by Reimann and colleagues brings some light to this puzzle [14]. The authors observed increased S100A4 expression in the vasculature of both human COPD and mouse lung. S100A4 is also known as a fibroblast specific protein – 1 (FSP-1), it is widely reported to be upregulated in fibrosis and cancer in different organs and is one of the key proteins which are upregulated during EMT [8, 39, 40]. We have previously reported that EMT is an active process in smokers and COPD with S100A4 as one of the key proteins up-regulated along with vimentin and others, and contributing to the fibroblasts and CAFs populations in COPD [5, 8, 9]. Increased S100A4 expression in pulmonary vasculature of COPD patients suggests that process of endothelium to mesenchymal transition (EndMT) is also active in COPD in addition to EMT. It is quite possible that the primary arterial smooth muscle cells staining positively for S100A4 are the transitioning endothelial cells [38], hence the process of EndMT is active in COPD contributing to pulmonary vascular remodelling [38]. EndMT might be the mechanism behind increased expression of S100A4 in COPD. Similar staining pattern for S100A4 was observed in vessels by our group as well when we analysed bronchial biopsies and surgically resected lung tissue from smokers and COPD for EMT related changes [5, 8, 9]. These are important observations and warrant further studies, as there are no studies in COPD so far looking for the evidence of active EndMT but it is quite possible that this process is active.

## Conclusions

EMT is likely the process which is contributing to small airway fibrosis and large airway epithelial cancers. Vascular remodelling is also a key feature of COPD but the mechanisms behind are poorly understood. In line with current literature and observations from the recent study by Reimann and colleagues it is quite possible that the process of EndMT is active in lungs of COPD patients. EndMT is possibly contributing to vascular remodelling in COPD BUT at the same time is also potentially contributing to airway fibrosis and malignancy by producing fibroblasts and cancer associated fibroblasts (CAFs), this warrants further studies. If this is true, it will have huge implications for understanding the underlying pathology of COPD. We believe, that understanding EMT and EndMT might be the key to understanding what COPD is really about and its nasty clinical consequences.

## Abbreviations

CAFs: Cancer associated fibroblasts
COPD: Chronic obstructive pulmonary disease
ECM: Extracellular matrix
EMT: Epithelial mesenchymal transition
EndMT: Endothelial to mesenchymal transition
LP: Lamina propria
Rbm: Reticular basement membrane
